# Effects of microbial agents on cadmium uptake in *Solanum nigrum* L. and rhizosphere microbial communities in cadmium-contaminated soil

**DOI:** 10.3389/fmicb.2022.1106254

**Published:** 2023-01-05

**Authors:** Meng You, Li Wang, Guopeng Zhou, Yikun Wang, Kai Wang, Rong Zou, Weidong Cao, Hongli Fan

**Affiliations:** ^1^Key Laboratory of Plant Nutrition and Fertilizer, National Engineering Research Center of Arable Land Protection, Ministry of Agriculture and Rural Affairs, Institute of Agricultural Resources and Regional Planning, Chinese Academy of Agricultural Sciences, Beijing, China; ^2^Institute of Soil and Fertilizer, Guizhou Academy of Agricultural Sciences, Guiyang, Guizhou, China; ^3^College of Forestry, Guizhou University, Guiyang, Guizhou, China

**Keywords:** phytoremediation, cadmium, *Solanum nigrum L.*, microbial agent, rhizosphere microbe

## Abstract

*Solanum nigrum* L. (*S*. *nigrum*) and microbial agents are often used for the remediation of cadmium (Cd)-contaminated soil; however, no studies to date have examined the efficacy of using various microbial agents for enhancing the remediation efficiency of Cd-contaminated soil by *S*. *nigrum*. Here, we conducted greenhouse pot experiments to evaluate the efficacy of applying *Bacillus megaterium* (BM) along with citric acid (BM + CA), *Glomus mosseae* (BM + GM), and *Piriformospora indica* (BM + PI) on the ability of *S*. *nigrum* to remediate Cd-contaminated soil. The results showed that BM + GM significantly increased the Cd accumulation of each pot of *S*. *nigrum* by 104% compared with the control. Application of microbial agents changed the soil microbial communities. Redundancy analysis showed that the activities of Catalase (CAT) and urease (UE), soil organic matter, available N and total Cd were the main influencing factors. By constructing the microbial co-occurrence networks, the soil microbe was divided into four main Modules. BM + GM and BM + PI significantly increased the relative abundance of Module#1 and Module#3, respectively, when compared with the control. Additionally, Module#1 showed a significant positive correlation with translocation factor (TF), which could be regarded as the key microbial taxa. Further research found that *Ascomycota*, *Glomeromycota*, *Proteobacteria*, and *Actinobacteria* within Module#1 were also significantly correlated with TF, and these key species enriched in BM + GM. Overall, our findings indicate that the BM + GM treatment was the most effective for the remediation of Cd pollution. This treatment method may further affect the rhizosphere microbial community by affecting soil indicators, which might drive the formation of Module#1, thus greatly enhancing the Cd remediation capacity of *S*. *nigrum*.

## 1. Introduction

Industrial activities are a major source of heavy metal (HM) pollution in farmland; mining, nickel-cadmium (Cd) battery manufacturing, and other anthropogenic activities are some of the major contributors to HM pollution, which has become a global environmental problem that affects more than 200 million hectares of arable land (Mani et al., 2015; Pramanik et al., 2018). Cd can have deleterious effects on the growth of plants, and Cd contamination can pose major risks to human health as it bioaccumulates in the food chain (Zhang et al., 2018). Cd has been classified as a hazardous chemical of global significance by the United Nations Environment Programme, and an increasing number of studies have examined the remediation of Cd-contaminated soils, especially in developing countries (Peng et al., 2014).

Physical, chemical and biological methods for remediation of cadmium pollution have various disadvantages, some of which include high costs, easy to produce secondary pollutants, and their inability to be applied for long periods (Baker et al., 2000). The phytoremediation is a cost-effective, eco-friendly and promising approach (Abdelkrim et al., 2020), this approach includes phytoextraction, rhizofiltration, phytostabilization and phytovolatilization (Saha et al., 2017). Among them, phytoextraction is based on the capacity of the roots to absorb, translocate, and concentrate toxic metals from soil to the shoot harvestable plant tissues (Girdhar et al., 2014). The use of accumulators is an effective method for the phytoextraction of HM-contaminated soils that is low-cost and environmentally friendly (Robinson et al., 1998; Fan and Zhou, 2009). *Solanum nigrum* L. (*S*. *nigrum*) grows rapidly and has a high reproductive capacity; its antioxidant defense system makes it highly resistant to HMs. However, some of the drawbacks of using *S*. *nigrum* are its long growing period and low environmental adaptability, which reduces its remediation efficiency (Wang L. et al., 2020; Khalid et al., 2021). The use of plant microbes and chelating agents along with *S*. *nigrum* can enhance the remediation efficiency of Cd-contaminated soil, and this approach has received increased interest from various researchers (Sheng et al., 2012).

Plant growth-promoting rhizobacteria (PGPR) are highly tolerant to Cd due to their physical isolation and detoxification capacity (Rasouli-Sadaghiani et al., 2019). When they are colonized in the rhizosphere of plants, they can assist plant to detoxify Cd by altering antioxidant enzyme activity and phytohormone secretion in plant (Ashraf et al., 2017), and promoting plant growth and development *via* nitrogen (N) fixation and siderophore production. *Bacillus megaterium* (Bm) is a common, aerobic, and gram-positive PGPR. A previous study showed that inoculation of Bm promoted production of organic acids to enhance the Cd bioavailability in soil, and promoting plant growth (Jeong et al., 2012). Additionally, it is safe to use on food crops and does not pose any harm to humans and animals. Thus, it is a promising microbe for promoting the efficiency of phytoremediation. Inoculation of arbuscular mycorrhizal fungi (AMF) is also a common measure to promote the efficiency of phytoremediation, which are widespread in ecosystem. They can colonize the roots of plants and form symbiosis with nearly 90% of land plant species. AMF thus play key roles in the cycling of plant nutrients, soil conservation, and plant–soil interactions. Some investigations have demonstrated that AMF can ameliorates growth of plants by enhancement of productivity and nutrient acquisition and increase Cd bioavailability in the soil, and mediate the absorption and transfer of metals (Luo et al., 2017; Wang G. et al., 2020). *Glomus mosseae* (Gm) is an AMF that can enhance the ability of plants to acquire nutrients and promote plant growth. And it can also alter the form of HMs to enhance the HM tolerance of host plants, thereby improving the remediation efficiency of HM pollution (Mani et al., 2015; Yang et al., 2016). Scientists in northwestern India discovered *Piriformospora indica* (Pi) in 1998 (Verma et al., 1998; Mao, 2016). Pi is a mycorrhizal-like fungus that is morphologically similar to AMF; it also has some of the same ecological roles as AMF and can enhance the resistance of plants to HM pollution by increasing the activity of antioxidant enzymes. Previous studies have shown that Pi plays a key role in stabilizing concentrations of Cd in the roots and decreasing concentrations of Cd in the stems and leaves (Shahabivand et al., 2017; Khalid et al., 2022; Li et al., 2022). In recent years, chelating agents have been employed to enhance the efficiency of phytoremediation in soil, because they have relatively strong complexing ability with HM (Huang G. et al., 2020). Among them, citric acid (Ca) can dissolve HMs in soil, enhance their bioavailability, and promote the absorption, transport, and enrichment of HMs by plants (Nowack et al., 2008). A previous study has shown that the addition of Ca increases root biomass and the concentration of Cd in *S*. *nigrum* (Rehman et al., 2017).

Several studies have identified functional strains that enhance plant growth and stress resistance in plant–microbe systems. Although many studies have evaluated the efficacy of using single microbial strains for HM-contaminated soil remediation by plants, few studies have evaluated the efficiency of using microbes combined with other microbes or low-molecular-weight organic acids for the remediation of Cd-contaminated soil. The results of recent studies indicate that the co-inoculation of several microbes can increase the dry weight and stem height of oat plants (*Avena sativa*; Xun et al., 2015). The concentration and removal rate of HMs are increased in Indian mustard following inoculation with Bm and Ca (Wang et al., 2014). The findings of these studies suggest that additional investigation of the effects of the application of several microbes or chelating agents on phytoremediation efficiency is needed. However, the nutrient uptake ability of plants can vary, and HMs might affect the relationships between microbes and host plants. In addition, HM tolerance varies greatly among plant species, even under the same stress conditions. The effects of microbes combined with other microbes or Ca on the efficiency of Cd remediation by *S*. *nigrum* have not yet been explored.

Here, we conducted pot experiments in a greenhouse to evaluate the effects of Bm combined with Ca, Gm, and Pi on the ability of *S*. *nigrum* to remediate Cd-contaminated soil. Our specific aims were to (1) characterize the effects of different additives on Cd accumulation in *S*. *nigrum*, (2) clarify the effects of different additives on the diversity and structure of rhizosphere microbial communities, (3) explore the relationship between changes in microbial structure and Cd-contaminated soil remediation by *S*. *nigrum*, and (4) identify the most optimal combination of different additives for the remediation of Cd pollution.

## 2. Materials and methods

### 2.1. Experimental materials and design

*Solanum nigrum*, Bm and Pi, and Gm were obtained from the Agro-environmental Protection Institution, Ministry of Agriculture and Rural Affairs; the Agricultural Culture Collection of China; and the Institute of Plant Nutrition, Resources and Environment, Beijing Academy of Agriculture and Forestry Sciences, respectively.

Cultivated soil (depth of 0–20 cm) was collected from farmland polluted with Cd in Anyang, Henan Province (36°05′N, 114°23′E) and used in the greenhouse pot experiments; the concentration of Cd in this soil was 2.12 mg/kg. The basic physical and chemical properties of the soil were as follows: pH, 8.26; soil organic matter (SOM), 28.2 g·kg^−1^; available N (AN), 115 mg·kg^−1^; available phosphorus (AP), 12.1 mg·kg^−1^; available potassium (AK), 222 mg·kg^−1^; cation exchange capacity (CEC), 12.5 cmol·kg^−1^; available Cd (ACd), 0.870 mg·kg^−1^; and total Cd (TCd), 2.12 mg·kg^−1^. The experiment comprised five treatments with three replicates for each treatment: *S*. *nigrum* (CK), Bm addition (BM), Bm + Ca (BM + CA), Bm + Gm (BM + GM), and Bm + Pi (BM + PI). In the BM and BM + CA treatments, 20 ml of Bm bacterial solution was added to each plot when plants were transplanted, and 20 ml of Bm and 10 mmol/kg Ca were added to each pot after 15 days. Gm (30 g) and Pi (5 ml) were added to each pot in the BM + GM and BM + PI treatments, respectively, when plants were transplanted, and Bm (20 ml) was added to each pot 15 days later.

*Solanum nigrum* seeds were treated with 0.5% (v/v) NaClO for 15 min; they were then washed with deionized water several times and placed in a constant-temperature water bath at 40°C for 6 h. The seeds were cultivated in a sterilized nutrient substrate, placed in a light incubator, and then transplanted to pots until *S*. *nigrum* plants had five leaves. A total of 1.5 kg of soil was added to each pot. A layer of soil was applied to cover the strains. No fertilizer was added, and plants were watered with distilled water once a week during the experiments. The experiments were performed in a greenhouse with an 8-h/16-h light/dark photoperiod (25/20°C day/night temperature) at 65–75% relative humidity. All plant and soil samples were collected after plants matured at 45 days of growth.

### 2.2. Collection of samples and measurements

Root and shoot samples of *S*. *nigrum* were collected and rinsed with tap water and deionized water, respectively. Cd^2+^ attached to the surface of roots was removed by immersing root samples in 20 mmol·L^−1^ Na_2_-EDTA for 15 min; these samples were then washed with deionized water several times. All plant samples were dried at 105°C for 30 min and then kept at 80°C until a constant weight was achieved. A ball mill was used to grind the samples; the samples were then filtered through a 0.25-mm sieve. HNO_3_ + HCIO_4_ (4:1) was used to digest the plant samples, and inductively coupled plasma mass spectrometry (ICP–MS; 7,700x, Agilent, Foster City, United States) was used to estimate Cd concentrations (Huo et al. 2018). Citrus leaves were used as the reference material [GBW10020 (GSB-11), 0.17 ± 0.02 mg·kg^−1^ Cd] for assessing the quality of the Cd concentration data.

The root shaking method was used to collect rhizosphere soil samples, and these samples were stored at –80°C for subsequent determination of soil microbial diversity. High-throughput sequencing was conducted by Shanghai Meiji Biotechnology Co., Ltd., and these data were used to estimate microbial diversity. According to the Closing Report from Majorbio, a FastDNA Spin Kit for Soil (MP Biomedicals, Santa Ana, CA, United States) was applied for sediment DNA extraction. Then, a NanoDrop 2000 spectrophotometer (Thermo Fisher Scientific, Wilmington, DE, United States) was used for the extracted DNA quantification. Before PCR amplification, all of the DNA samples were stored at –80°C. The bacterial and fungal communities in different samples were analyzed using Illumina MiSeq sequencing. The primers 338F (5′-ACTCCTACGGGAGGCAGCAG-3′)/806R (5′-GGACTACHVGGGTWTCTAAT-3′) and ITS1F (5′-CTTGGTCATTTAGAGGAAGTAA-3′)/ITS2R (5′-GCTGCGTTCTTCATCGATG C-3′) were used to amplify the V3–V4 region of the bacterial 16S rRNA gene and the fungal ITS1 region, respectively. All the forward primers were tagged with a 5-nucleotide barcode to distinguish different samples. After verification by agarose gel electrophoresis, the purification of amplicons was processed with EZNA Cycle-Pure Kit (Omega Bio-tek Inc., Doraville, GA, United States). The purified amplicons were next pooled by normalizing in equimolar numbers and subjected to high-throughput sequencing (Illumina MiSeq PE300 platform; Majorbio Bio-pharm Technology, Shanghai, China). All analyses were performed in triplicate.

After crushing the remaining air-dried soil samples, they were filtered through 2-mm and 0.25-mm sieves for subsequent determination of the TCd and available ACd content of the soil and the physical and chemical properties of the soil. Sulfuric acid, nitric acid, and hydrogen peroxide were used for the microwave digestion samples, and the TCd concentration was measured using ICP–MS. Diethylene triamine pentaacetic acid (DTPA) was used to extract ACd, and ICP–MS (7,700X, Agilent, United States) was used to measure the concentration of ACd. Various kits (Suzhou Keming Biotech, China) were used to determine the activity of catalase (CAT), urease (UE), and alkaline phosphatase (ALP) in the soil samples. The methods described in a previous study were used to determine the basic physical and chemical properties of the soil samples (Xu et al., 2020). The ammonium acetate method (1 M CH_3_COONH_4_) at pH 7.0 was used to determine the CEC.

### 2.3. Data analysis

SPSS 20 was used to conduct statistical analyses. Analysis of variance (ANOVA), followed by Duncan’s tests, was used to evaluate the significance of differences between groups; the threshold for statistical significance was *p* < 0.05. Figures of the data were created using Origin (2019) software:

1. Bioaccumulation factor (BCF) = Cd concentration in the shoot/TCd concentration in soil.
2. Translocation factor (TF) = Cd concentration in the shoot/Cd concentration in the roots.

[Cd] indicates the content of Cd in a specific part of the plant in mg·kg^−1^.

The Majorbio Cloud platform^1^ was used to create a series of maps of microbial diversity and community composition. The R packages “WGCNA” and “igraph” were used to conduct symbiotic network analysis, and 15 samples with a combined number of operational taxonomic units (OTUs) of less than 150 were excluded from analyses. A total of 638 bacterial and 167 fungal OTUs were used to construct nodes and edge files for the symbiotic network graph. Gephi software was used to divide the taxa in the network into different modules using default parameters. The main modules were retained, and they were visualized and highlighted in different colors using Gephi. The OTUs of each Module were zero-mean normalized, and correlation analyses of sample means with the BCF and TF of shoots were performed to identify the key modules. Correlations between bacterial genera and the BCF and TF of shoots in the key Modules were determined to identify the bacteria associated with the BCF and TF.

## 3. Results

### 3.1. Uptake and transport of cadmium in *Solanum nigrum*

**Figure 1:**
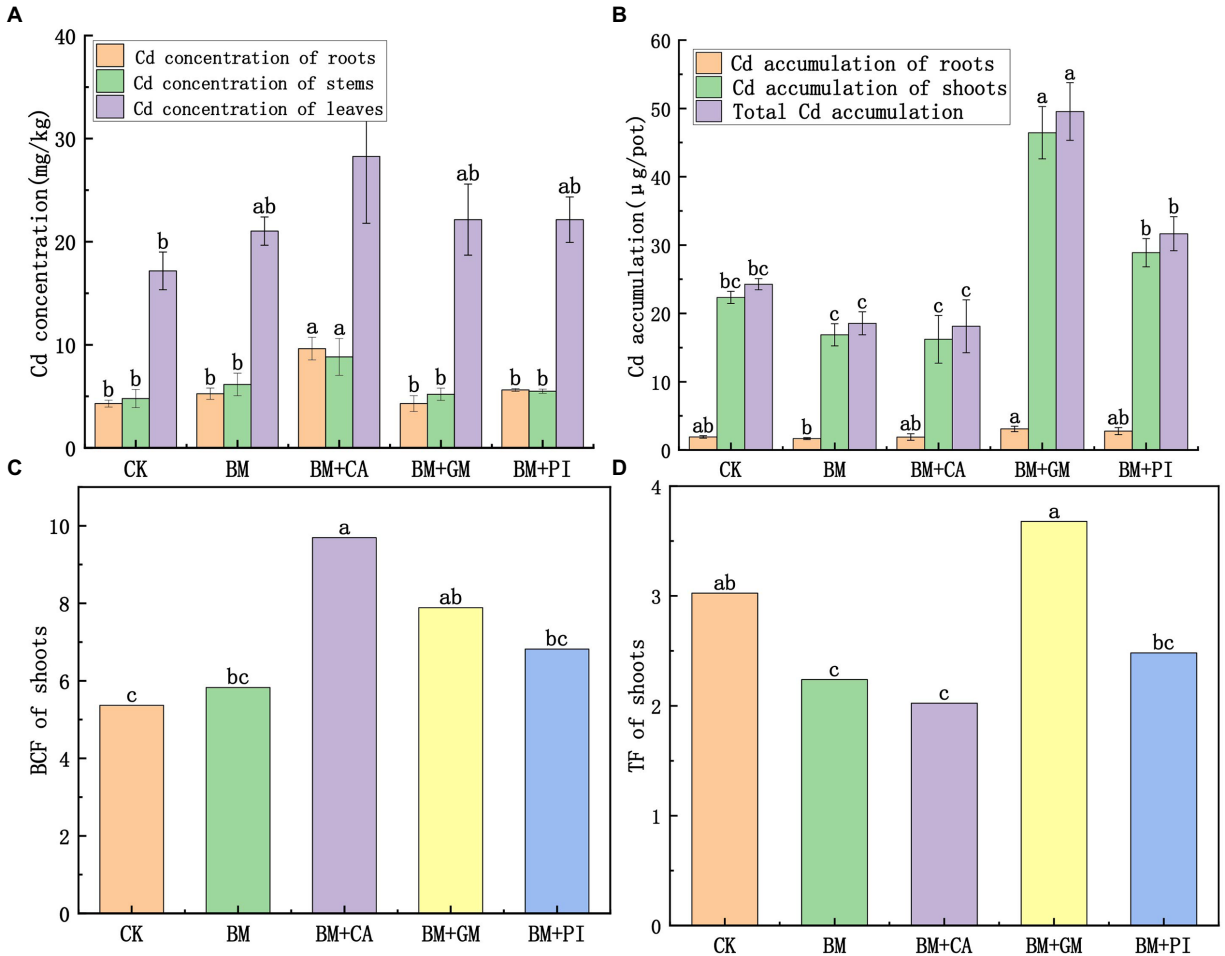
The effects of different treatments (*S*. *nigrum* (CK), *Bacillus megaterium* (BM) along with citric acid (BM + CA), *Glomus mosseae* (BM + GM), and *Piriformospora indica* (BM + PI)) on the Cd concentrations **(A)** and accumulation of Cd **(B)** in *S*. *nigrum*. Variation in BCF and TF of the shoots of *S*. *nigrum* in different treatments **(C,D)**. Error bars show the standard deviation of triplicate samples, and columns with different letters are significantly different according to one-way ANOVA, followed by Duncan’s test (*p* < 0.05).

The Cd concentrations in the root, stem, and leaf tissues of *S*. *nigrum* were 124, 84.4, and 64.6% higher in the BM + CA treatment (Figure 1A) than in the CK, and these differences were significant. The BCF of *S*. *nigrum* shoots was 80.6% higher in the BM + CA treatment than in the CK (Figure 1C). There were no significant differences in Cd accumulation in the roots, shoots, and entire *S*. *nigrum* plants between the BM + CA treatment and the CK (Figure 1B). The TF of *S*. *nigrum* shoots was 33.1% lower in the BM + CA treatment than in the CK (Figure 1D). The Cd concentration in each part of *S*. *nigrum* was higher in the BM + GM treatment than in the CK (Figure 1A), but these differences were not significant. The accumulation of Cd in each part of *S*. *nigrum* was higher in the BM + GM treatment than in the CK; Cd accumulation was 108 and 104% higher in the shoots and entire plants in the BM + GM treatment than in the CK, respectively, and these differences were significant (Figure 1B). The BCF and TF of *S*. *nigrum* shoots was 46.9 and 21.6% higher in the BM + GM treatment than in the CK, respectively (Figures 1C, D); the difference in BCF between the BM + GM treatment and the CK was significant. These findings indicate that the Cd uptake and accumulation capacity of *S*. *nigrum* were higher in the BM + GM treatment than in the CK.

### 3.2. Microbial diversity and community composition in the rhizosphere

The Ace and Chao indices of bacteria varied among the BM, BM + CA, and CK treatments; specifically, the Ace and Chao indices of bacteria were greater in the BM treatment than in the CK and lower in the BM + CA treatment than in the CK, which suggests that the BM and BM + CA treatments had opposite effects on the species richness of bacteria (Table 1). The Ace and Chao indices of fungi were higher in the BM and BM + CA treatments than in the CK. These findings indicate that the BM and BM + CA treatments had a significant effect on the species richness of bacteria and fungi. Similar findings were obtained from Venn diagrams (Supplementary Figure S1).

**Table 1:** Diversity indexes of bacteria and fungi in different treatments.

	Bacteria			Fungi		
Treatments	Shannon	Ace	Chao1	Shannon	Ace	Chao1
CK	6.67 ± 0.02a	3,754 ± 124ab	3,776 ± 138ab	3.00 ± 0.41a	518.2 ± 8.1a	509.0 ± 6.2a
BM	6.77 ± 0.05a	3,903 ± 107a	3,929 ± 109a	3.07 ± 0.72a	544.2 ± 50.0a	553.1 ± 48.1a
BM + CA	6.59 ± 0.13a	3,387 ± 246b	3,402 ± 251b	2.98 ± 0.47a	562.5 ± 54.0a	569.0 ± 49.4a
BM + GM	6.71 ± 0.03a	3,613 ± 77ab	3,683 ± 65ab	3.32 ± 0.24a	511.7 ± 15.2a	520.0 ± 27.6a
BM + PI	6.69 ± 0.07a	3,742 ± 243ab	3,803 ± 287ab	2.71 ± 0.41a	510.4 ± 46.4a	518.3 ± 57.4a

CK, BM, BM + CA, BM + GM, BM + PI represent *S*. *nigrum*, *Bacillus megaterium*, and *Bacillus megaterium* along with citric acid, *Glomus mosseae*, and *Piriformospora indica*, respectively. The error bars show the standard deviation of triplicate samples, and values with different letters are significantly different according to one-way ANOVA followed by Duncan’s test (*P* < 0.05).

**Figure 2:**
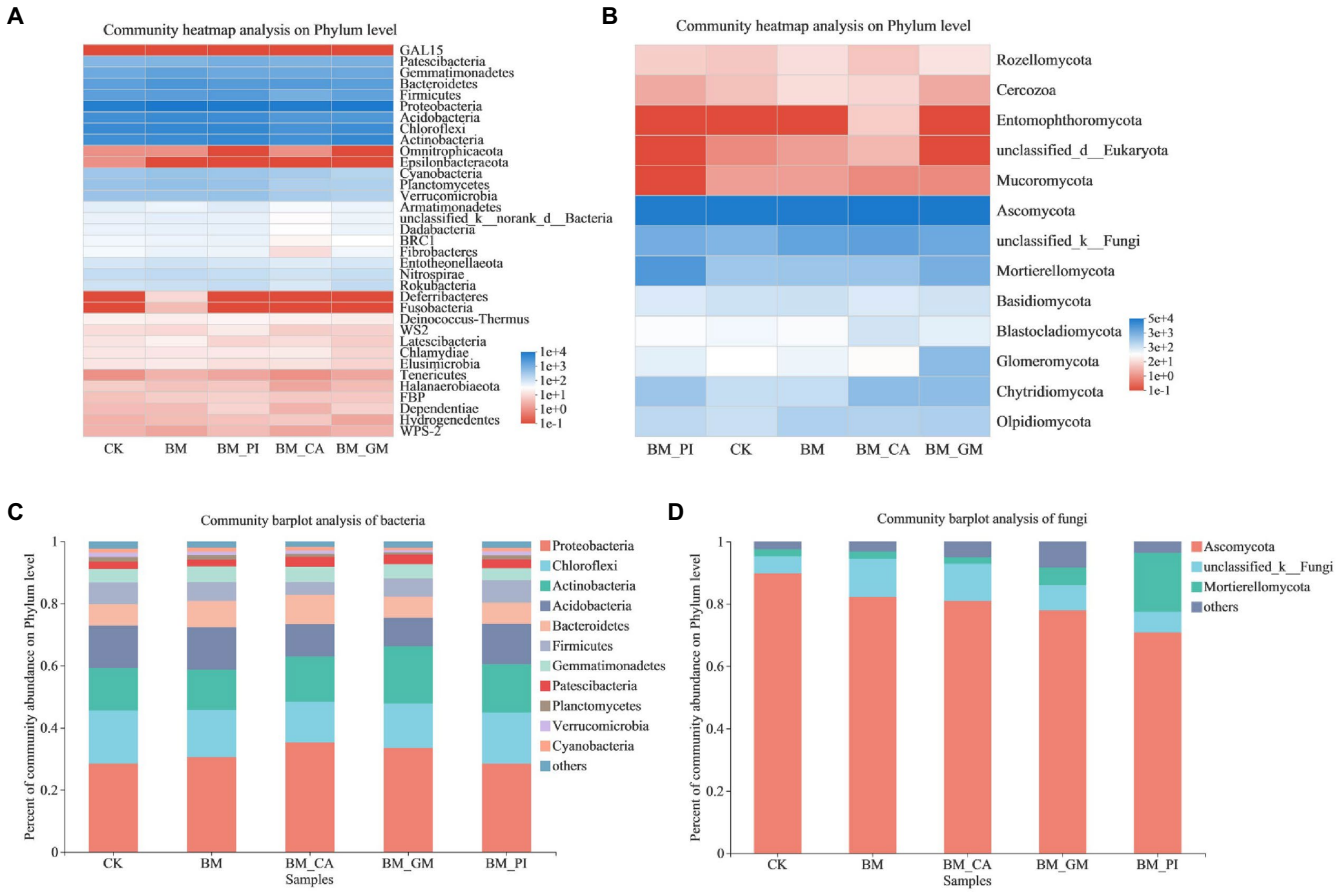
The community heatmap analysis **(A,B)** and abundances of communities **(C,D)** of bacteria and fungi in the different treatments [*S*. *nigrum* (CK), *Bacillus megaterium* (BM) along with citric acid (BM + CA), *Glomus mosseae* (BM + GM), and *Piriformospora indica* (BM + PI)].

We conducted community heatmap analysis and community bar plot analysis of bacteria and fungi to clarify the composition of microbial species in the soil. The relative richness of *Proteobacteria* was the highest among all bacterial communities. The relative richness of *Patescibacteria*, *Gemmatimonadetes*, *Bacteroidetes*, *Firmicutes*, *Acidobacteria*, *Chloroflexi*, and *Actinobacteria* was also high (Figure 2A). The dominant fungal phyla were *Ascomycota*, and *Mortierellomycota*, and the abundance of *Glomeromycota* was significantly higher in the BM + GM treatment than in the CK (Figure 2B). The abundances of *Proteobacteria* and *Actinobacteria* were higher in the BM + GM treatment than in the CK (Figure 2C). The relative abundance of *MortieRellomycot* was higher in the BM + GM and BM + PI treatments than in the CK (Figure 2D).

**Figure 3:**
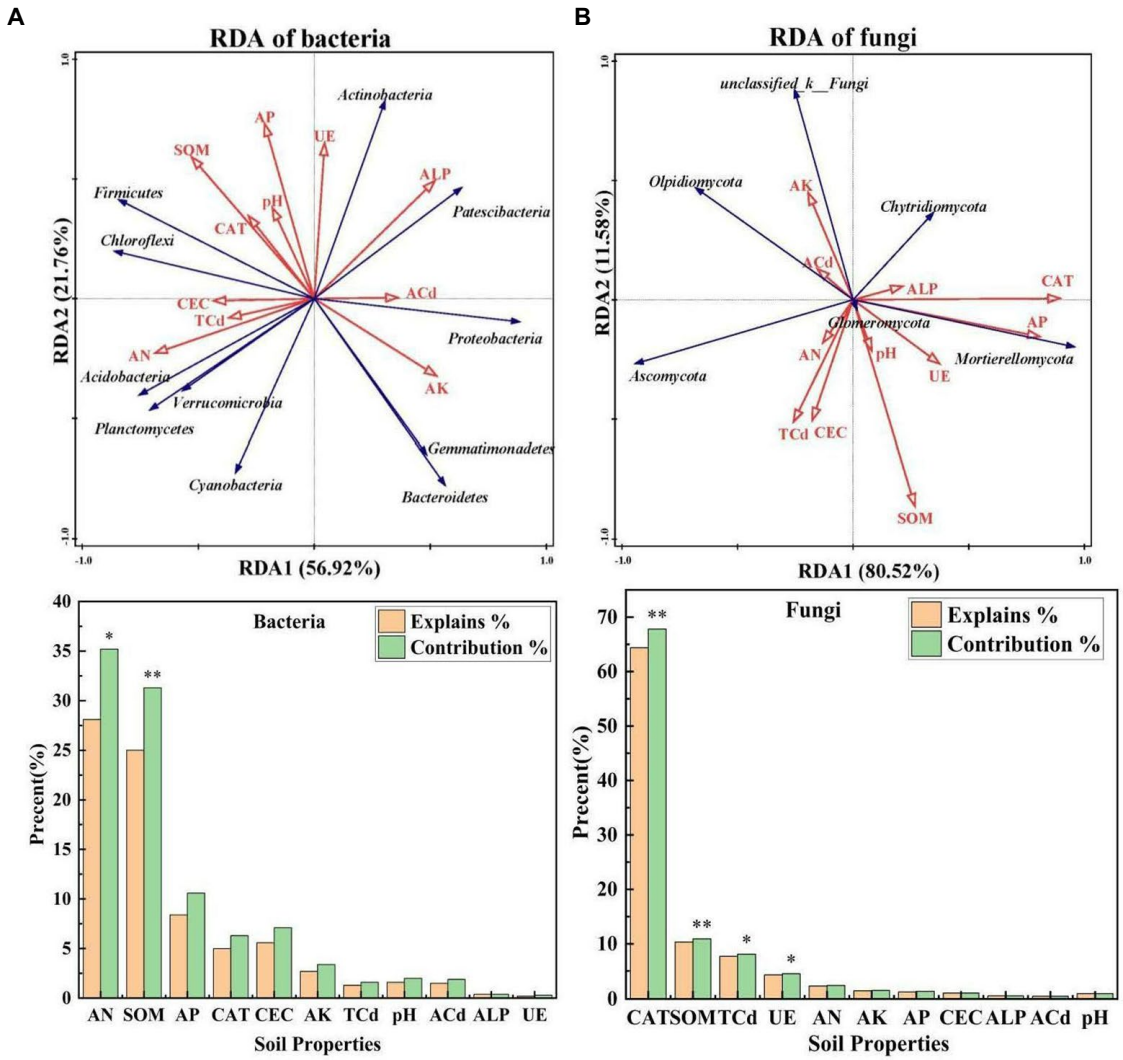
**(A,B)** Redundancy analysis of the dominant bacteria, dominant fungi, and environmental factors under different treatments [*S*. *nigrum* (CK), *Bacillus megaterium* (BM) along with citric acid (BM + CA), *Glomus mosseae* (BM + GM), and *Piriformospora indica* (BM + PI)]. The blue arrows indicate microbial communities, and the red arrows indicate soil properties. ALP, CAT, and UE indicate alkaline phosphatase activity, catalase activity, and urease activity, respectively; AN, AP, and AK indicate available nitrogen, available phosphorus, and available potassium, respectively; SOM and CEC indicate soil organic matter and cation exchange capacity, respectively; and TCd and ACd indicate total cadmium and available cadmium, respectively. Values marked with * indicate significant differences at *p* < 0.05, and values marked with ** indicate significant differences at *p* < 0.01.

RDA1 explained 56.92 and 80.52% of the total variation in bacterial and fungal communities, and RDA2 explained 21.76 and 11.58% of the total variation in bacterial and fungal communities, respectively (Figures 3A, B), which suggests that soil properties had a major effect on the structure of microbial communities. AN and SOM explained 28.1 and 25.0% of the change in bacterial communities, respectively, and their effects on the composition of bacterial communities were significant (*p* < 0.05). CAT, SOM, TCd, and UE explained 64.4, 10.3, 7.70, and 4.30% of the change in fungal communities, respectively, suggesting that they were the key environmental factors affecting the composition of fungal communities (*p* < 0.05). The correlations of bacterial and fungal communities with soil properties also varied.

### 3.3. Network analysis and keystone microbial taxa

**Figure 4:**
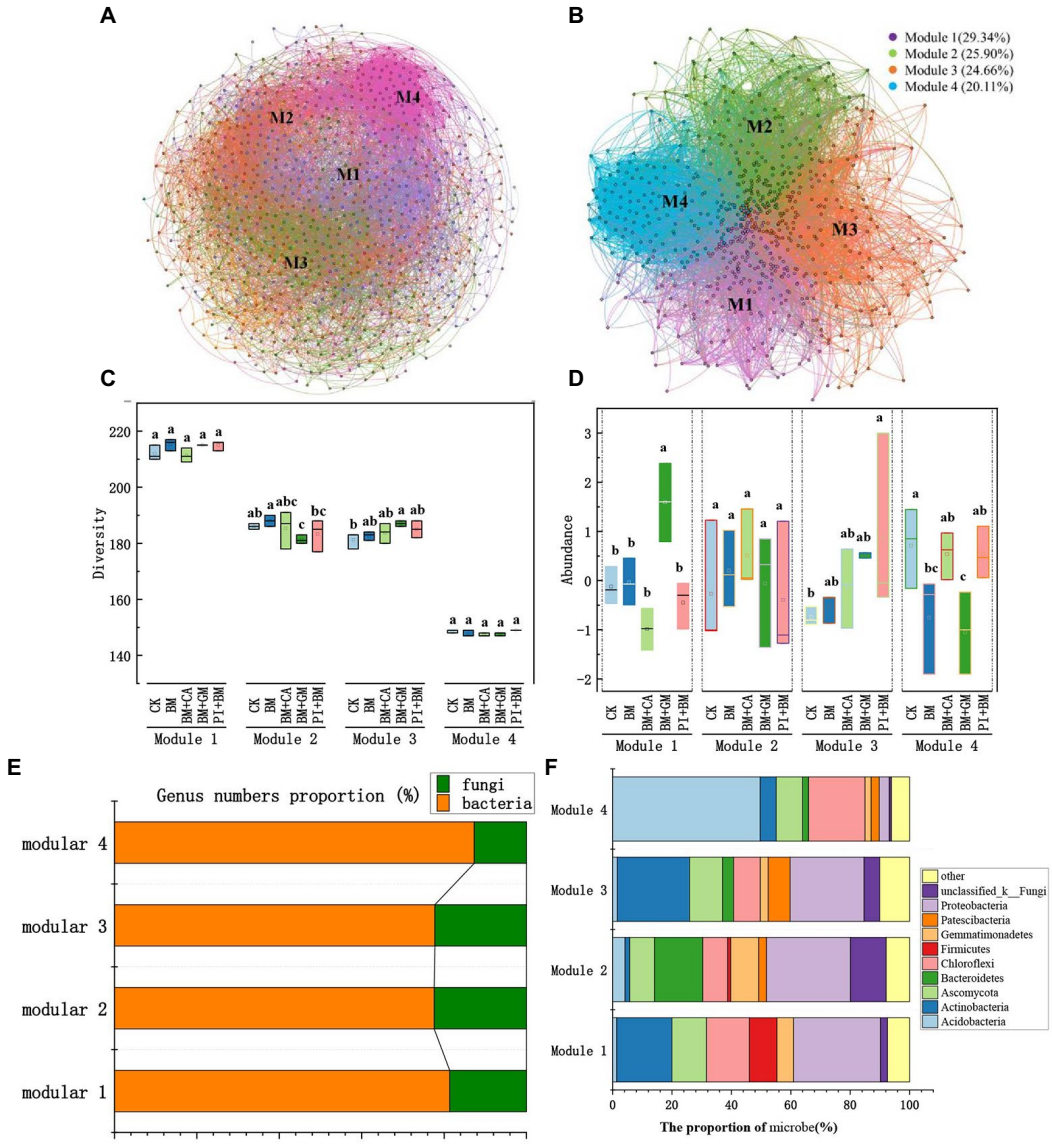
Keystone microbial taxa. **(A)** Network map of bacterial and fungal genera; microbial kingdoms in the network are indicated by the colors of the node. **(B)** Network map of microbial taxa from modules 1–4; modules in the network are indicated by different colors. **(C)** Microbial diversity in modules 1–4. **(D)** Microbial abundance in modules 1–4; the data were zero-mean normalized. **(E,F)** Composition of microbes in modules 1–4. CK BM BM + CA BM + GM BM + PI represent *S*. *nigrum*, *Bacillus megaterium*, and *Bacillus megaterium* along with citric acid, *Glomus mosseae*, and *Piriformospora indica*, respectively. The error bars show the standard deviation in triplicate samples, and values with different letters significantly differ according to one-way ANOVA followed by Duncan’s test.

We constructed a bacterial and fungal network to identify key microbial groups (Figure 4A). The network was divided into four major microbial taxa, which were referred to as modules 1 to 4, and these four modules accounted for 93.98% of the total nodes. After other modules were removed, we constructed a microbial network for modules 1, 2, 3, and 4 (Figure 4B), which accounted for 29.34, 25.90, 24.66, and 20.11% of the nodes, respectively. Modules 1–4 all comprised bacteria and fungi, but the relative abundances of bacteria and fungi in each Module differed (Module#1: 81.36% bacteria and 18.64% fungi; Module#2: 77.60% bacteria and 22.40% fungi; Module#3: 77.78% bacteria and 22.22% fungi; and Module#4: 87.33% bacteria and 12.67% fungi; Figure 4E). Microbial diversity in Module#3 was significantly higher in the BM + GM treatment than in the CK; however, microbial diversity was significantly lower in Module#2 in the BM + CA treatment than in the CK (Figure 4C). The abundances of microbes were significantly higher in Module#1 in the BM + GM treatment and in Module#3 in the BM + PI treatment than in the CK (Figure 4D). We also compared variation in the composition of microbes among modules (Figure 4F). There was substantial variation in the composition of microbes among modules; the dominant bacteria and fungi previously mentioned accounted for 92.56, 92.15, 89.95, and 93.88% of all microbes in Module#1, 2, 3, and 4, respectively (Figures 2C, D). *Actinobacteria*, *Ascomycota*, *Chloroflexi*, and *Proteobacteria* accounted for 18.60, 11.63, 14.42, and 29.30% of all microbes in Module#1, respectively. *Bacteroidetes*, *Proteobacteria*, and unclassified _ k _ Fungi accounted for 16.23, 28.27, and 12.04% of all microbes in Module#2, respectively. *Actinobacteria*, *Ascomycota*, and *Proteobacteria* accounted for 24.34, 11.11, and 24.87% of the microbes in Module#3, respectively. In Module#4, *Acidobacteria* and *Chloroflexi* accounted for 49.66 and 19.05% of all.

### 3.4. Associations of keystone microbial taxa with Bioaccumulation factor and translocation factor

**Figure 5:**
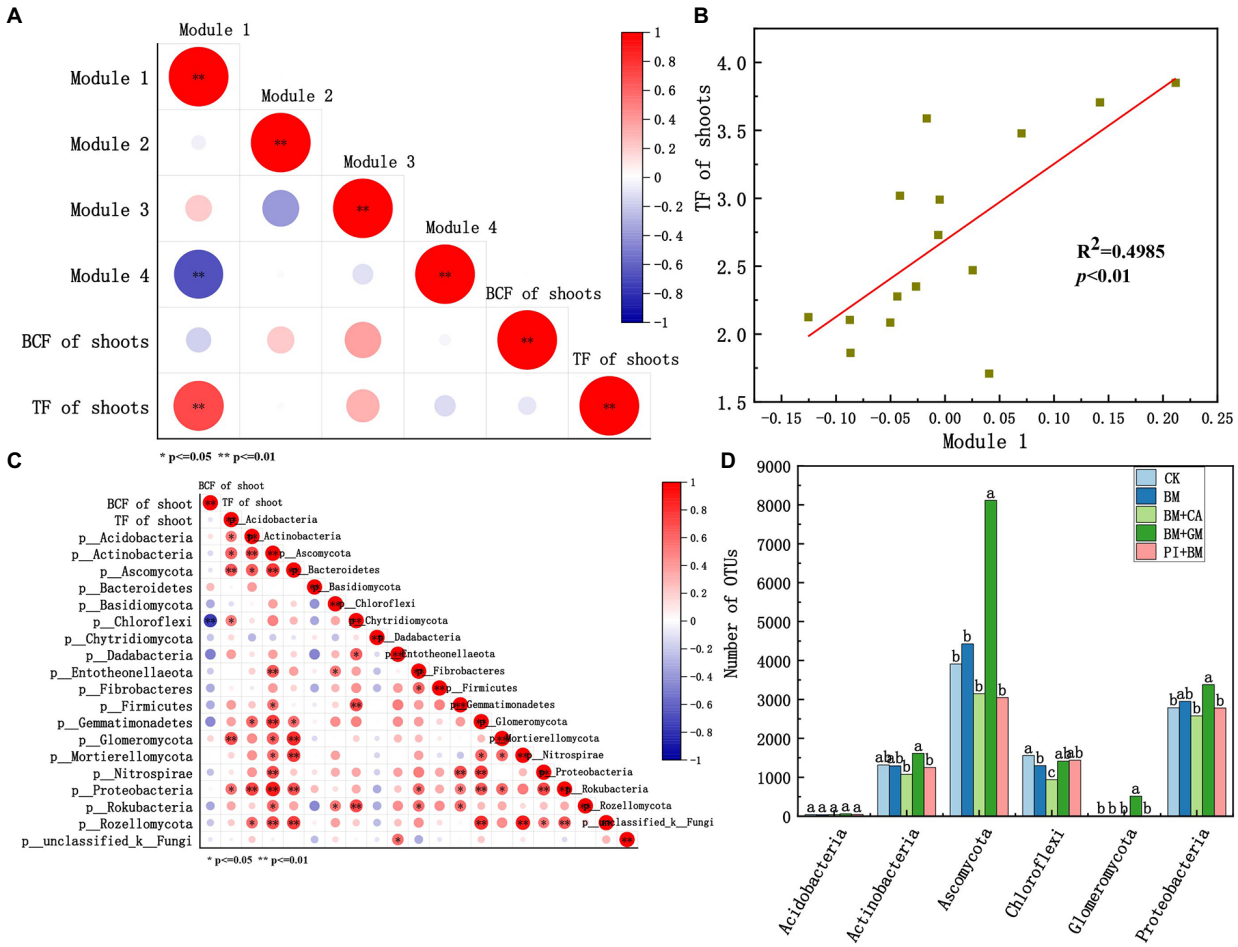
**(A)** Correlations of modules 1–4 with the BCF and TF of the shoots according to Pearson correlation analysis. **(B)** Linear regression analysis between Module#1 and the TF of shoots. **(C)** Correlation of microbes in Module#1 with the TF of shoots according to Pearson correlation analysis. **(D)** Number of bacterial OTUs in Module#1. Asterisks indicate statistically significant differences according to Student’s t-test (*p < 0.05, **p < 0.01). CK BM BM + CA BM + GM BM + PI represent *S*. *nigrum*, *Bacillus megaterium*, and *Bacillus megaterium* along with citric acid, *Glomus mosseae*, and *Piriformospora indica*, respectively.

We evaluated relationships of the standardized mean OTU values of microbial taxa (modules 1–4) with the BCF and TF of shoots (Figure 5A). Module#1 was significantly positively correlated with the TF of shoots, and the coefficient of determination of this correlation was high (R^2^ = 0.4985) (Figure 5B). Thus, Module#1 comprises key microbial taxa in our study. *Acidobacteria*, *Actinobacteria*, *Ascomycota*, *Chloroflexi*, *Glomeromycota*, and *Proteobacteria* in Module#1 were significantly positively correlated with the TF of shoots (Figure 5C). An ANOVA of the number of bacterial and fungal OTUs in different treatments was conducted. The number of *Ascomycota*, *Glomeromycota*, *Proteobacteria*, and *Actinobacteria* OTUs was significantly higher in the BM + GM treatment than in the CK (Figure 5D), suggesting that these taxa might enhance the translocation of HMs in *S*. *nigrum*.

## 4. Discussion

Phytoremediation is an environmentally friendly and widely used approach to remediate HM-contaminated soil (Yan et al., 2020). Plant biomass is an important factor that affects the success of phytoremediation. Both plant biomass and the concentration of HMs in plants affect the efficiency of phytoremediation (Zhiguo and Qixing, 2009; Jeong et al., 2012; Hou et al., 2017). Soil microbes are abundant and play key roles in the growth and development of plants (Kloepper and Schroth, 1978); however, their precise effects depend on the plant species, microbial species, and the type of HM (Khalid et al., 2021). Bm is a typical PGPR, and previous studies indicate that Bm can secrete organic acids, such as indole acetic acid, to activate Cd and acidify soil (Jeong et al., 2012; Huang H. et al., 2020). In our study, the accumulation of Cd in *S*. *nigrum* was lower in the BM than in the CK treatment. This might stem from the activation of Cd due to the secretion of organic acids by Bm; this excess Cd is then absorbed by *S*. *nigrum*, which causes the concentration of Cd in *S*. *nigrum* to exceed the range that it can tolerate. Consequently, the growth of *S*. *nigrum* was inhibited following exposure to high levels of Cd, and the biomass (Supplementary Figure S2) and accumulation of Cd were lower in *S*. *nigrum* in the BM treatment than in the CK. The activity of HM ions is increased by Ca, as it reduces the pH of soil and promotes the absorption of N, P, K, and other nutrient elements by plants. Thus, Ca can promote the growth and development of plants and the absorption of HM ions (Huang G. et al., 2020; Han R. et al., 2021). The findings of our study indicate that the combined application of Bm and Ca significantly decreased the biomass of *S*. *nigrum*. The Cd concentration was significantly higher in the BM + CA treatment than in the CK and the accumulation of Cd was lower in the BM + CA treatment than in the CK; this might stem from the increase in the bioavailability of Cd, which suppressed the growth of *S*. *nigrum*. The fact that the Cd concentration in *S*. *nigrum* was highest in the BM + CA treatment is also consistent with these findings. Gm is a common arbuscular mycorrhizal fungus, and Pi is a mycorrhizal-like fungus; they have been previously shown to enhance the resistance of plants to HMs (Dabral et al., 2019). In our study, the biomass of *S*. *nigrum* was significantly higher in the BM + GM treatment than in the CK; although the Cd concentration in *S*. *nigrum* was only slightly higher in the BM + GM treatment than in the CK, Cd accumulation was significantly higher in the BM + GM treatment than in the CK. This finding indicates that the concentrations of HMs were diluted in *S*. *nigrum* in the BM + GM treatment, and this primarily stemmed from increases in the growth and biomass of *S*. *nigrum*, which enhanced the remediation efficiency of Cd-contaminated soil (Yang et al., 2021). Bm can activate a large number of Cd ions and increase the concentrations of N, P, K, and other nutrient elements, which increases the uptake of Cd and nutrient elements by *S*. *nigrum*. However, Gm alleviated the toxicity of Cd to *S*. *nigrum*, which enhanced its growth; this indicates that BM and GM had synergistic effects. Pi promotes the growth of plants under both normal and stress conditions (Verma et al., 1998). Pi mitigates the deleterious effects of oxidative stress by enhancing the antioxidant defense system and regulating antioxidant levels in plants, which increases the resistance of plants to stress (Vadassery et al., 2009). Pi has been used to mitigate HM stress in wheat, sunflower, *Nicotiana tabacum* L., and other plants (Shahabivand et al., 2012; Hui et al., 2015; Shahabivand et al., 2017). Previous studies have shown that Pi can increase the yield of *Hordeum vulgare* L. and *Brassica napus* L.; it can also enhance tolerance to water stains and the production of *Brassica pekinensis* (Lour.) Rupr (Waller et al., 2005). Following inoculation with Pi, the resistance of *Arabidopsis thaliana* seedlings to drought is significantly enhanced (Sherameti et al., 2008). The results of our study indicate that co-inoculation of Bm and Pi increased the biomass of *S*. *nigrum* and increased Cd accumulation in *S*. *nigrum*, which suggests that Pi enhanced the resistance of *S*. *nigrum* to the deleterious effects of Cd; however, the protective effects of Gm were stronger than those of Pi.

During the phytoremediation of HM-contaminated soil, plants absorb HMs from the soil and the HMs are transported from the roots to the shoots is a key component. The phytoremediation efficiency can be evaluated according to three criteria: the tolerance of plants to HMs, the ability of plants to absorb HMs, and the ability of plants to transport HMs from the roots to the shoots (Rajkumar et al., 2009; Chen et al., 2014). BCF is an indicator of the ability of plants to absorb HMs from soil; plants with higher BCF values have a stronger ability to absorb and accumulate HMs (Zhou X. et al., 2020; Liu et al., 2022). In our study, the BCF was higher in the four treatments than in the CK, suggesting that the uptake of Cd in *S*. *nigrum* was promoted in the four treatments. The most significant increase in Cd uptake was observed in the BM + CA treatment, which suggests that the BM + CA treatment had the most significant effect on the efficacy of phytoremediation. This might be explained by the fact that Bm plays an important role in promoting the activation of soil Cd; Ca also promotes the activation of soil Cd (Marques et al., 2013; Yang et al., 2022). TF is an indicator of the ability of plants to transport HMs from the roots to the shoots (Chi et al., 2022). In our study, the TF of *S*. *nigrum* was significantly lower in the BM and BM + CA treatments than in the CK. The TF of *S*. *nigrum* was significantly higher in the BM + GM treatment than in the CK. There was no difference in the TF of *S*. *nigrum* between the BM + PI treatment and the CK. Some studies have indicated that Cd is stabilized by AMF through mycelia or *via* adsorption by chitin cell walls, which impedes the transport of HMs from the roots to the shoots; this regulates the distribution of HMs in plants, reduces the toxicity of HMs, and promotes plant growth (Christie et al., 2004; Wang et al., 2007; Hui et al., 2015; Wu et al., 2015). The inoculation of Pi under Cd stress limits the movement of HMs to the shoots of the plant and promotes the accumulation of Cd in the roots. Fungi can mitigate the toxicity of HMs in host plants (Hui et al., 2015; Shahabivand et al., 2017). The results of our study indicate that the ability of *S*. *nigrum* to transport Cd from the roots to the shoots was enhanced in the BM + GM treatment compared with the CK; this might stem from the fact that the toxicity of Cd was reduced and the growth of *S*. *nigrum* was promoted in the presence of Gm which could lead to higher phytoextraction efficiency (Han Y. et al., 2021). The effects of the BM + PI and BM + GM treatments on *S*. *nigrum* might be similar. The protective effects of Pi were weaker than those of Gm, so the ability of *S*. *nigrum* to transport HMs was weaker in the BM + PI treatment than in the BM + GM treatment. High concentrations of Cd had a toxic effect on *S*. *nigrum* in the BM and BM + CA treatments, and this affected the normal physiological metabolism of *S*. *nigrum*. Although the roots absorbed a large number of Cd ions, they were not transported to the shoots.

Phytoremediation is a promising technique for the remediation of HM-contaminated soils, and it might affect the diversity and structure of rhizosphere microbes. During the remediation process, the rhizosphere microbial community plays a role in energy transfer, nutrient circulation, HM resistance, and HM detoxification in soil (Guo et al., 2019). We conducted various analyses to clarify the effects of different treatments on rhizosphere microbes. The effects of different treatments on fungi and bacteria varied, which suggests that changes in rhizosphere microbes are affected by changes in the surrounding environment. *Proteobacteria*, *Acidobacteria*, *Actinobacteria*, and *Bacteroidetes* are the most common bacterial phyla in the rhizosphere (Guo et al., 2019). In our study, *Patescibacteria*, *Chloroflexi*, *Actinobacteria*, *Acidobacteria*, and *Bacteroidetes* were the dominant bacterial phyla, and *Ascomycota*, unclassified _ k _ fungi, *Mortierellomycota*, *Glomomycota*, *Chytridiomycota*, and *Oldiomycota* were the dominant fungal phyla. Variation in the abundances of bacteria might stem from variation in soil type; the factors that explain variation in the abundance of fungi remain unclear because few relevant studies have been conducted. The most significant differences in bacteria and fungi among treatments were observed in *Proteobacteria*, *Actinobacteria*, *Ascomycota*, unclassified _ k _ fungi, and *MortieRellomycot*. Some studies have shown that *Proteobacteria* are highly resistant to HM pollution because they are capable of adapting to environments with high concentrations of HMs (Spain et al., 2009; Wang X. et al., 2021). In our study, the relative abundance of *Proteobacteria* was higher in the BM + CA and BM + GM treatments than in the CK; consequently, the concentrations of Cd were higher in the BM + CA and BM + GM treatments than in the CK. The accumulation of Cd was particularly pronounced in *S*. *nigrum* in the BM + GM treatment, and the concentration of Cd in the roots of *S*. *nigrum* was particularly high in the BM + CA treatment. *Actinobacteria* can enhance the resistance of plants to disease (Tang et al., 2016). In our study, the relative abundance of *Actinobacteria* was highest in the BM + GM treatment, which provided further support for this conclusion. Other studies have explored the relationship between CAT activity and the resistance of plants to disease. CAT can mitigate damage induced by Cd stress by scavenging reactive oxygen species (Pramanik et al., 2018; Khalid et al., 2022); thus, CAT might explain why *Actinobacteria* enhance the disease resistance of plants. *MortieRellomycot* has been shown to alleviate the deleterious effects of stress and increase the bioavailability of HMs (Xun et al., 2017). In our study, the abundance of *MortieRellomycot* was significantly higher in the BM + PI treatment than in the other treatments; this might explain why Cd accumulation was higher in the BM + PI treatment. Other dominant microbial communities play key roles in soils; for example, *Acidobacteria* play an important role in the decomposition of organic matter in poor soils, and *Bacteroidetes* mediate the decomposition of macromolecular organic matter (Naumoff and Dedysh, 2012; Rawat et al., 2012). Thus, changes in rhizosphere microbes during the remediation of contaminated soils can be marked. Various environmental factors affect the relative abundances of bacteria and fungi. In our study, AN and SOM were the main factors affecting the relative abundances of bacteria, and CAT, SOM, TCd, and UE were the main factors affecting the relative abundances of fungi; this suggests that microbial diversity and community composition were mainly affected by environmental factors in the rhizosphere.

Microbial taxa constructed based on the microbial co-occurrence networks reflect habitat heterogeneity, different selection regimes and phylogenetically closely related species clusters (Shi et al., 2016), which were shown to be composed of interactions between co-existing microbial species and plant-induced changes in nutrient status, HM behavior, etc. in the immediate soil environment (Chen et al., 2018; Hassani et al., 2018). The microbial co-occurrence networks that we established contained four main modules that differed significantly in microbial composition, and the percentage distribution of bacterial and fungal phyla varied considerably among modules. Variation in the diversity of OTUs among modules was not as pronounced as variation in the abundances of microbial taxa among modules; this indicates that environmental factors had a greater effect on the abundances of microbes rather than the composition of microbes. The abundances of OTUs in Module#1 were significantly higher in the BM + GM treatment than in the CK, and the abundances of OTUs in Module#3 were significantly higher in the BM + PI treatment than in the CK. OTUs in Module#1 were considered key microbial taxa given that there was a significant correlation between Module#1 and shoot TF. *Acidobacteria*, *Actinobacteria*, *Ascomycota*, *Chloroflexi*, *Glomeromycota*, and *Proteobacteria* in Module#1 were also significantly positively correlated with shoot TF. Given that Cd accumulation and the OTUs numbers of *Ascomycota*, *Glomeromycota*, *Proteobacteria*, and Actinobacteria were higher in the BM + GM treatment than in the CK, these taxa likely enhance the ability of *S*. *nigrum* to transport Cd. In fact, *Proteobacteria*, *Actinobacteria* have been shown to have high adaptability to Cd and protective ability for plants (DeAngelis et al., 2009; Luo et al., 2021), which suggests that they might contribute to the hyperaccumulation of Cd in plants. *Glomeromycota* could also significantly increase plant resistance and biomass under HM stress condition (Amna et al., 2015). Similarly, *Ascomycota* might enhance the ability of plants to absorb Cd, as well as plant growth. The specific effects need to be further studied and added. It is worth mentioning that *Glomeromycota*, although not a dominant fungal phylum, its abundance in BM + GM treatment was particularly noticeable. It was likely to be a change caused by Gm, which may be related to the improvement of host tolerance by Gm.

In our study, high Cd concentrations were a major stress for *S*. *nigrum* and soil microbes. Under stress conditions, plants engage in cooperative interactions with microbes in the environment, and this results in changes in the rhizosphere environment, which in turn alters the structure of microbial communities (Liu et al., 2020; Tang et al., 2020). Only microorganisms that can tolerate Cd stress are able to survive, and microbial communities might affect plants by affecting phytohormones, signaling, and nutrient acquisition, which can alter the efficiency of phytoremediation (Yergeau et al., 2014; Zhou F. et al., 2020; Wang G. et al., 2021). In our study, Module#1 taxa played a key role in phytoremediation, and a network of functionally complementary microbes was formed that enhanced phytoremediation efficiency. The BM + GM treatment might drive the formation of this network, which greatly enhanced the Cd remediation capacity of *S*. *nigrum*.

## 5. Conclusion

In Cd-contaminated soil, co-inoculation of Bm and Gm significantly enhanced the growth of *S*. *nigrum* and the accumulation of Cd. The inoculation of different agents further influenced the soil microbial community by affecting soil AN, CAT, SOM, TCd, and UE. By constructing the microbial co-occurrence networks, the soil microbe was divided into four main Modules. BM + GM and BM + PI significantly increased the relative abundance of Module#1 and Module#3, respectively, when compared with the control. Additionally, Module#1 was considered as the key microbial taxon by response to TF of *S*. *nigrum*, in which *Ascomycota*, *Glomeromycota*, *Proteobacteria*, *Actinobacteria* were identified in close relationship with TF of *S*. *nigrum*, demonstrating their ability to enhance phytoremediation efficiency. Totally, Bm and Gm can be used for phytoremediation of Cd-contaminated soils, and the importance of key microbial taxa in phytoremediation was emphasized. Additional studies are needed to clarify the mechanisms underlying the interactions among *S*. *nigrum*, microbes, and soil properties in HM-contaminated soils.

## Data availability statement

The datasets presented in this study can be found in online repositories. The names of the repository/repositories and accession number(s) can be found at: https://www.ncbi.nlm.nih.gov/, PRJNA905327; https://www.ncbi.nlm.nih.gov/, PRJNA905569.

## Author contributions

MY: conceptualization, investigation, validation, methodology, formal analysis, writing-original draft, and writing-review and editing. LW: conceptualization, resources, data curation, and performing the experiment. GZ and YW: software and writing-review and editing. KW: resources, data curation, and software. RZ: resources, data curation, and performing the experiment. WC: supervision, project administration, and writing-review and editing. HF: supervision, funding acquisition, project administration, and writing-review and editing. All authors contributed to the article and approved the submitted version.

## Funding

This work was supported by the National Key Research and Development Program of China (Nos. 2021YFD1700200 and 2016YFD0800800).

## Conflict of interest

The authors declare that the research was conducted in the absence of any commercial or financial relationships that could be construed as a potential conflict of interest.

## Publisher’s note

All claims expressed in this article are solely those of the authors and do not necessarily represent those of their affiliated organizations, or those of the publisher, the editors and the reviewers. Any product that may be evaluated in this article, or claim that may be made by its manufacturer, is not guaranteed or endorsed by the publisher.

## Footnotes

^1^ www.majorbio.com
