# Supplementary material for: Effects of microbial agents on cadmium uptake in Solanum nigrum L. and rhizosphere microbial communities in cadmium-contaminated soil
Source: Front Microbiol. 2023 Jan 5;13:1106254. doi: 10.3389/fmicb.2022.1106254 (PMC9849675; doi:10.3389/fmicb.2022.1106254)
Supplement: Supplementary file 1 [file Data_Sheet_1.PDF]

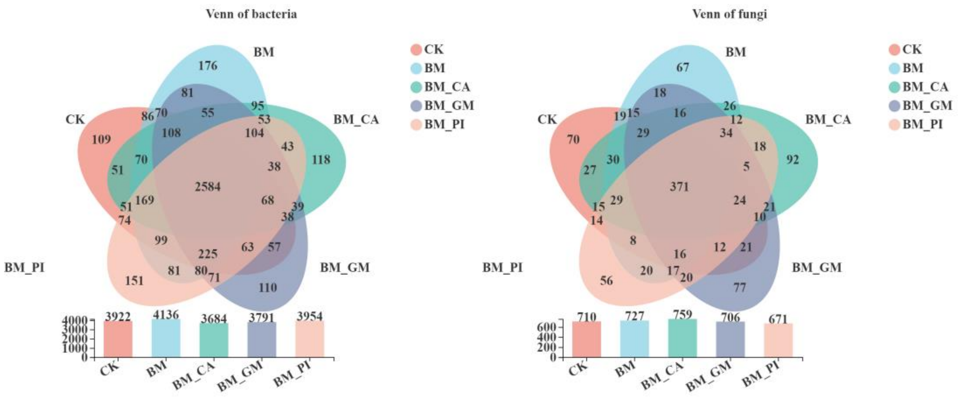

Supplementary Figure 1. OTU Venn diagram of bacteria and fungi under different treatments.

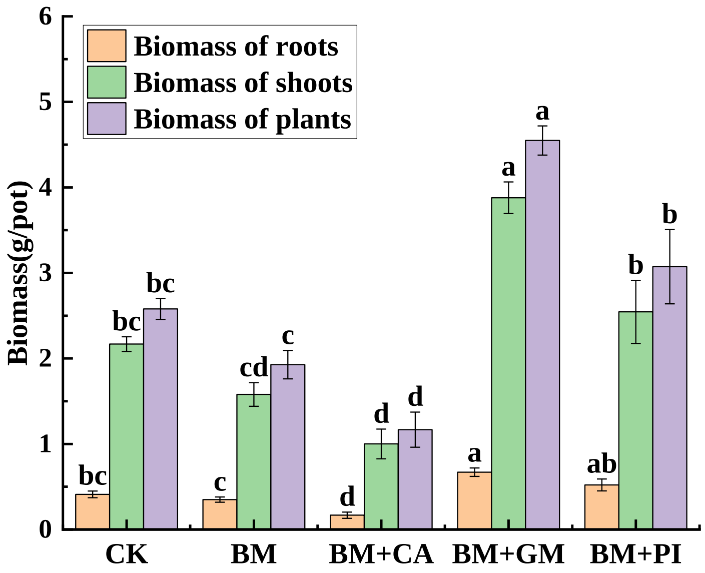

Supplementary Figure 2.The effect of different treatments on the biomass. The error bars show the calculated standard deviation in triplicate samples, and values with different letters significantly differ by one-way ANOVA and Duncan's test (P < 0.05).

Table1 Effects of different treatments on soil properties.

Treatments	PH	Organic matter （g·kg-1）	Available N （mg·kg-1）	Available P （g·kg-1）	Available K （mg·kg-1）	CEC （cmol·kg-1）
CK	8.31±0.14a	26.1±0.2a	100±1.72b	23.2±1.53b	323±17.6a	14.0±1.76a
BM	8.31±0.09a	24.4±0.29b	103±1.09a	23.9±0.41b	336±13.7a	13.0±0.18a
BM+CA	8.21±0.02a	24.6±0.68b	94.1±1.65c	22.6±0.56b	391±44.7a	12.6±0.25a
BM+GM	8.37±0.09a	25.5±0.29a	98.2±0.73b	26.4±1.15a	328±41.8a	12.9±0.03a
BM+PI	8.31±0.05a	25.8±0.26a	98.8±1.67b	27.7±0.8a	320±33.1a	13.1±0.23a

Table2 Effects of different treatments on soil total Cd, effective Cd concentrations and enzyme activity.

Treatments	ACd (mg·kg-1)	TCd (mg·kg-1)	CAT （umol/d/g）	UE (ug/d/g)	ALP (umol/d/g)
CK	0.83±0.05a	2.46±0.53a	58.08±2.91a	547.4±61.2ab	9.43±3.37ab
BM	0.81±0.08a	2.02±0.15a	61.44±0.34a	460.9±94.9b	6.64±2.96b
BM+CA	0.87±0.02a	2.02±0.22a	59.96±1.54a	521.4±76.5ab	12.29±0.14a
BM+GM	0.84±0.07a	2.00±0.15a	61.69±1.11a	618.4±49.2a	12.63±0.77a
BM+PI	0.81±0.03a	2.04±0.04a	69.38±1.86a	598.9±32.3a	10.78±1.13ab
